# Integrating short- and full-length 16S rRNA gene sequencing to elucidate microbiome profiles in Pacific white shrimp (*Litopenaeus vannamei*) ponds

**DOI:** 10.1128/spectrum.00965-24

**Published:** 2024-09-27

**Authors:** Dora M. Rajonhson, Pacharaporn Angthong, Timpika Thepsuwan, Sawannee Sutheeworapong, Penpicha Satanwat, Paveena Tapaneeyaworawong, Sorawit Powtongsook, Worarat Kruasuwan, Piroon Jenjaroenpun, Thidathip Wongsurawat, Sage Chaiyapechara, Wanilada Rungrassamee

**Affiliations:** 1 National Center for Genetic Engineering and Biotechnology, National Science and Technology Development Agency, Khlong Luang, Pathum Thani, Thailand; 2 Pilot Plant Development and Training Institute, King Mongkut’s University of Technology Thonburi, Bangkok, Thailand; 3 Center of Excellence for Marine Biotechnology, Department of Marine Science, Faculty of Science, Chulalongkorn University, Bangkok, Thailand; 4 Division of Medical Bioinformatics, Research Department, Faculty of Medicine Siriraj Hospital, Mahidol University, Bangkok, Thailand; 5 Siriraj Long-read Lab (Si-LoL), Faculty of Medicine Siriraj Hospital, Mahidol University, Bangkok, Thailand; Dominican University New York, Orangeburg, New York, USA

**Keywords:** intestinal microbiota, environmental microbiota, *Litopenaeus vannamei*, short-read sequencing, full-length 16S rRNA gene sequencing

## Abstract

Despite their immense economic value as a key aquaculture species, the production of Pacific white shrimp (*Litopenaeus vannamei*) faces significant challenges from intensive farming practices and disease outbreaks. Routine microbial profiling for disease surveillance could be a promising approach to anticipate and control disease outbreaks. To achieve this, accuracy in microbial profiling in shrimp ponds is crucial for enabling targeted action and prevention. Extensive documentation emphasizes that, beyond biological factors (related to the host, diet, or health status during the rearing period), technical elements, including sequencing techniques significantly influence bacterial community profiling. This study investigated the influence of short- and long-read sequencing of 16S rRNA genes on the microbial profiles in shrimp intestines, water, and sediments. The origin of the samples (intestine or environmental) in shrimp culture ponds primarily drove the observed differences in core microbial species. The ecological niches accounted for 56% of bacterial community variations in culture ponds. Both sequencing approaches showed consistent results in identifying higher-rank taxa and assessing alpha and beta diversity. However, at the species level, full-length 16S rRNA gene sequences provided better resolution than V3–V4 sequences. For routine microbial profiling in shrimp culture ponds, our study suggests that short-read sequences were sufficient for determining overall bacterial community.

**IMPORTANCE:**

This interdisciplinary study investigated the influence of sequencing techniques on bacterial communities profiling within Pacific white shrimp (*Litopenaeus vannamei*) ponds. By integrating aquaculture, microbiology, and environmental science, we revealed the role of ecological niches and factors like salinity and pH on microbiota diversity and composition in shrimp intestines, pond water, and sediment. Additionally, we compared the taxonomic resolution using partial versus full-length 16S rRNA gene sequences, highlighting the value of longer amplicons for precise identification of key taxa. These findings provide novel insights into microbial dynamics underlying environmental effects in shrimp aquaculture. Comprehensive characterization of the pond microbiome could lead to management strategies that promote shrimp health and productivity. Furthermore, the potential of a multi-omics approach for integrating complementary data streams to elucidate environment-microbiome-host interactions was highlighted.

## INTRODUCTION

*Litopenaeus vannamei*, commonly known as the Pacific white shrimp, is a crustacean species of economic significance due to its extensive global aquaculture production. The Pacific white shrimp industry served as a key driver of economic growth and employement opportunities (1, 2). With an annual market worth billions of dollars, primarily fueled by high international demand, the Pacific white shimp is projected to continue expanding in the coming decade (1). In the face of challenges associated with intensive farming practices and disease threats, shrimp microbiota emerged as a crucial player in disease prevention and immune system regulation (3).

The farming pond ecosystem represents a complex environment where microorganisms in water, sediment, and the intestine of the cultured animals interact—impacting growth, survival, nutrient cycling, and disease occurrence. The microbiota in these three units collectively contribute to the overall ecological balance in the ponds, ultimately supporting the growth of the cultured organisms. Intestinal microbiota aids in nutrient uptake, development, and health, while environmental (sediment and water) microbiota plays a role in water quality and nutrient cycling (4). Thus, the rearing environment is a critical area where cultivated shrimp are susceptible to contracting infections from pathogenic bacteria. An increase in the abundance of certain bacterial groups, resulting in a shift in the balance of the pond ecosystem, can have several negative consequences, These consequences include increased proliferation of pathogens and potential disease outbreaks (5). For instance, luminescent *Vibrio* in the pond has been known to cause severe shrimp mortalities (6). Furthermore, opportunistic pathogenic bacteria such as *Aeromonas*, *Flavobacterium*, and *Pseudomonas* can be present in the rearing environment (7). Hence, maintaining a healthy bacterial community in the rearing environment is vital to prevent the proliferation of pathogenic bacteria. A balanced microbial community is maintained by optimal water quality conditions and key environmental factors, such as temperature, pH, dissolved oxygen level, turbidity, and transparency (4, 7, 8).

Sediments in shrimp culture ponds accumulate due to leftover feed and fecal materials, resulting in a shared microbiota between the shrimp intestine and sediment (9). Sulfate-reducing bacteria in these sediments can produce hydrogen sulfide, which can have a detrimental effect on the rearing environment and shrimp health (10). The biofloc system, a common type of closed aquaculture system, facilitates the recycling of waste nutrients and the reduction of water usage (4, 11). This system necessitates controlled carbohydrate additions to promote the development of dense and active biofloc in the water (11), illustrating how the aquatic environment can be managed to cultivate beneficial microorganisms. Monitoring the microbial communities, including potential pathogens, within aquaculture systems provides valuable insights into the health status of cultured shrimp. This knowledge can inform targeted interventions aimed at optimizing shrimp health and productivity. Such interventions may include adjustments to water quality parameters, modifications to feed composition, or the implementation of probiotic treatments (12).

Key bacterial taxa, often referred to as “core” species, are frequently found in the intestinal microbiota of marine shrimp. This includes the prominent Pseudomonadota phylum, encompassing both beneficial and pathogenic bacteria like *Vibrio*, *Photobacterium*, and *Shewanella*. Bacillota, another significant phylum, hosts beneficial bacteria like *Bacillus* (13–15). Actinomycetota, Bacteroidota, and Cyanobacteria are also commonly encountered in *L. vannamei*’s intestinal microbiota, including genera like *Flavobacterium* and *Actinomyces* (13–18). The rearing environment of *L. vannamei*, including sediment and water in shrimp farming systems, harbors a diverse array of bacteria. Water contains Pseudomonadota, Bacillota, Bacteroidota, Actinomycetota, and Cyanobacteria, some of which can be beneficial or potentially pathogenic (19). *Vibrio* and *Ruegeria*, belonging to the Pseudomonadota phylum, are frequently detected in the water (16, 20, 21).

Most studies focusing on microbial community screening in shrimp culture ponds utilize high-throughput sequencing with amplicon-based approaches that target specific variable (V) regions of the bacterial 16S rRNA gene (22–25). Extensive documentation emphasizes that beyond biological factors (related to the host, diet, or health status during the rearing period), technical elements, including sequencing techniques and data analysis methods significantly influence bacterial community profiling (18). Among these technological considerations, sequencing the full-length and partial regions of the 16S rRNA gene can impact the taxonomic resolution, species-level identification, and advantageous for complementary information—an aspect that remains relatively unexplored in shrimp culture ponds. The choice between short-reads and long-reads in bacterial community profiling carries both advantages and limitations. Short-read 16S gene analyses offer cost-effective next-generation sequencing but are confined to genus-level resolution due to sequencing only a portion of the gene (26), limiting the distinction between highly similar species. On the other hand, full-length 16S sequences have the potential for species-level accuracy. Yet, long-reads come with higher sequencing error rates. Ongoing efforts aim to develop and optimize taxonomic identification algorithms capable of handling the increased read length and error rate associated with long-read data (27).

In this study, we focused on characterizing the bacterial communities present in the intestines of Pacific white shrimp (*L. vannamei*) and their rearing environment (water and sediment), using both short- and full-length 16S rRNA sequencing in their ponds. This integrated approach allowed us to identify and quantify the bacterial taxa present in each microbiome with high resolution and to explore the relationship between the intestinal microbiome and the environmental microbiomes (water and sediment) in shrimp aquaculture ponds. This integrated platform not only allowed us to assess the accuracy and resolution of microbial profiling in shrimp microbiome studies but also provided valuable insights into the interplay between environmental factors and the dynamics of microbial populations within aquaculture systems. Ultimately, these insights pave the way for optimizing shrimp rearing practices and achieving sustainable aquaculture.

## MATERIALS AND METHODS

### Shrimp rearing conditions and sample collection

Shrimp intestine (*n* = 10/pond), sediment (*n* = 4/pond), and water (*n* = 4/pond) samples were collected from two PPE-lined earthen ponds at Farm Song Nam, Chachoeng Sao (13°42′27.4″N, 101°04′53.9″E). The pond size was approximately 1,600 m^2^ with a 1.2 m depth, and the water salinity was in a range of ~10–12 ppt for Pond A and Pond B, respectively. Chlorination with approximately 10 ppm calcium hypochlorite was used for pond disinfection prior to use. After disinfection, ponds were left for 1 week before releasing shrimp. The specific pathogen-free *L. vannamei* shrimps, obtained from SyAqua Siam Co., Ltd, Thailand, were stocked at the density of approximately 70,000 shrimp/pond or 43 shrimp/m^2^. Feeding was performed with commercial shrimp feed (CPF, Thailand) with 38% protein at approximately 3% feed per shrimp weight per day using an automatic shrimp feeding machine. Feeding was continuously operated during daytime from 08:00 a.m. to 06:00 p.m. In Pond B, a probiotic containing a mixture of the three *Bacillus* species (*B. subtilis* BSN1, *B. megaterium*, and *B. licheniformis*), kindly provided by the Department of Fisheries (Thailand), was added following the instructions. Briefly, the commercial mix was inoculated in a 500 L tank with pond water and molasses and incubated for 24 h before being added to Pond B. The direct probiotic addition to the pond was carried out every 2 days. In parallel, the commercial probiotics were mixed with the feed pellets before being fed to the shrimp. The shrimp culture procedure in Pond A was similar but without the commercial probiotic supplement. Alkalinity (ALK) in both ponds was maintained at higher than 120 mg/L by adding sodium bicarbonate. Shrimp cultivation periods were 70 days for Pond A and 73 days for Pond B. During this period, shrimp body weight was recorded to assess weight gain, which the average weight of shrimp in the probiotic group was 19.05 ± 2.19 g, while those in the non-probiotic group averaged 16.65 ± 2.41 g (Table S1). During the crop, water quality parameters, including ALK, salinity, calcium chloride (CaCl_2_), magnesium chloride (MgCl_2_), nitrogen dioxide (NO_2_), ammonium (NH_4_), and pH, were monitored using colorimetric test kits and detected by a portable spectrophotometer (Spectrokit, Marine Leader, Thailand). ALK was monitored with a titration test kit (Aqua-Vac, Thailand).

All uses of animals in this experiment were approved by the National Center of Genetic Engineering and Biotechnology IACUC (Project code BT-Animal 24/2561).

### DNA extraction, purification, and short-read 16S rRNA gene sequencing

The individual whole shrimp intestine (approximately 150 mg) was extracted using the QIAamp DNA Mini Kit (Qiagen, Germany) following the manufacturer’s instructions (the reagent was scaled up to three reactions/sample). For DNA extraction from water and sediment, the 0.22 µm filter membrane containing bacterial cells from the water sample (100 mL each) and 250 mg of sediments were placed in 5 and 2 mL tubes, respectively, containing extraction buffer from the DNeasy PowerSoil Pro Kit (Qiagen, Germany) to extract DNA following the manufacturer’s instructions. All DNA samples were purified and concentrated using the Genomic DNA Clean and Concentrator (Zymo Research, USA) according to the manufacturer’s instructions. The purity and concentration of purified DNA were measured using a NanoDrop spectrophotometer (ND-8000, ThermoFisher Scientific, USA), and the concentration was confirmed with the Qubit dsDNA BR Assay Kit (Invitrogen, USA). Additionally, the quality of DNA samples was assessed by Agarose gel electrophoresis and stored at −80°C until further use.

DNA from shrimp intestines, water, and sediments was subjected to 16S rRNA gene amplification using primers (341F and 806R) targeting the V3–V4 variable regions of the 16S rRNA gene. Only 7 out of 10 intestine samples from Pond A had sufficient DNA yield for sequencing. The steps were carried out according to Illumina’s instructions. The polymerase chain reactions (PCRs) were conducted with Q5 high-fidelity DNA polymerase (New England Biolabs Inc., USA) using the following parameters: an initial denaturation at 94°C for 3 min, followed by 25 cycles of denaturation at 94°C for 30 s, annealing at 57°C for 45 s, extension at 72°C for 45 s, and a final extension at 72°C for 5 min. For each sample, PCR was performed in four replicates, pooled together, and then purified using the QIAquick gel extraction kit (Qiagen, USA). Subsequently, the purified DNA samples were sent for 16S rRNA Illumina MiSeq sequencing services (Omics Drive PTE. Ltd, Singapore).

Sequences were 250 bp paired-end reads in the FASTQ format. The data were processed using QIIME2-2022.2 (28). Adapters and primers were removed from both forward and reverse reads using q2-cutadapt with default parameters. Sequences were truncated at specified forward and reverse positions. Subsequently, paired-end reads were merged, handling overlapping regions, resulting in representative sequences of V3–V4 data. Chimeric sequences were identified and discarded using q2-dada2. Preprocessed sequences were clustered into features based on the amplicon sequence variant method. Feature counts per sample were organized into an abundance table (29) and were taxonomically annotated based on SILVA version 138 (30). To ensure comparability, rarefying normalization was applied, adjusting all samples to an equal number of reads. Finally, taxonomic abundances for all samples were calculated using q2-classify-sklearn.

### Full-length 16S rRNA gene library construction and sequencing

The full-length 16S rRNA gene was amplified using an universal 27F and 1492R primers (31), with an expected amplicon of ~1,500 bp. PCR was processed in a 100 µL total volume by using Q5 high-fidelity DNA polymerase (New England Biolabs Inc., USA) under the following conditions: initial denaturation for 30 s at 98°C; 25 cycles of denaturation for 10 s at 98°C; annealing for 30 s at 54°C; extension for 45 s at 72°C; and a final extension step for 2 min at 72°C. Subsequently, the amplicon (~200 ng) of each sample was subjected for library preparation using ligation sequencing amplicon—native barcoding kit (SQK-NBD114.96, Oxford Nanopore Technologies; ONT, Oxford, UK). The library was loaded into the MinION R10.4.1 flow cell (FLO-MIN114; ONT, Oxford, UK) and sequenced using GridION with the default setting. Base calling, adapter trimming, and demultiplexing were executed using Guppy version 6.5.7 in its super accuracy (SUP) mode. During the data cleansing phase, NanoFilt was employed to retain only the 16S rRNA gene sequences that were near complete (32). Sequences that were either shorter than 1,000 bp or longer than 2,000 bp, or those with a Q-score below 10, were discarded. Subsequently, the refined FASTQ files were processed using Emu software (27) in order to categorize 16S rRNA gene sequences taxonomically and to determine species-level relative abundances and read counts. Taxonomic classifications provided by Emu, incorporating both names and identifiers, were obtained from the DADA2 SILVA database, version 138.1, at the species level.

### Analysis of partial and full-length 16S rRNA gene sequences in shrimp intestines and environmental samples

Absolute abundance tables including domain, phylum, class, order, family, genus, and species were obtained. To ensure uniformity across samples, absolute abundances were rarefied and normalized using the R vegan package version 2.6.4, resulting in an equal number of reads for all samples. Subsequently, relative abundance tables were derived from the normalized absolute abundance tables. Taxonomic abundance profiles at the phylum, genus, and species levels were generated using the R ggplot2 package version 3.4.4. Alpha diversity, as measured by Shannon’s diversity index, observed features, and Pielou index, was estimated using the R vegan package version 2.6.4. To compare alpha diversity between Pond A (without probiotic supplementation) and Pond B (with probiotic supplementation) under each sample categories, boxplots, and statistical tests, including the Kruskal-Wallis and Wilcoxon Rank-Sum tests, were employed. Beta diversity, assessed through Bray-Curtis’s distance, was calculated and subsequently visualized by Principal Coordinate Analysis (PCoA) using R vegan package version 2.6.4 and phyloseq package version 1.46.0. Statistical testing for differences in beta diversity between groups and within groups was conducted using the Adonis test, also known as Permutational Multivariate Analysis of Variance Using Distance Matrices (PERMANOVA), with the R vegan package version 2.6.4. Differential abundant taxa between Pond A and Pond B under each sample type was identified using the R DESeq2 package version 1.42.0.

### Functional potentials of shrimp microbiomes and correlation network analysis

Full-length 16S rRNA gene sequences were extracted from the Emu database using species-level taxonomic classifications and their corresponding abundances to ensure accurate sequence-to-taxa matching, crucial for precise prediction of functional capabilities. Metabolic pathway predictions were conducted using the comprehensive Phylogenetic Investigation of Communities by Reconstruction of Unobserved States (PICRUSt2) pipeline v2.5.2 (33), executed through the picrust2_pipeline.py script with default parameters. This streamlined approach facilitated all essential steps for metabolic pathway prediction. Pathway predictions were visualized using pathway_errorbar from ggpicrust2 v1.7.3 (34) to assess the relative abundances and potential pathway activities of microbial communities. Plots detailed pathway distributions and highlighted significant variations (with Benjamini-Hochberg adjusted *P*-value less than 0.05) between sample groups, establishing the functional potential of microbial communities from 16S rRNA gene data and enhancing the understanding of their metabolic capabilities in different environments, specifically comparing bacterial communities between Pond A (without probiotic supplementation) and Pond B (with probiotic supplementation).

Correlation matrices, computed based on the relative abundance of the top 20 genera in each pond, were used to construct a Spearman’s correlation-based network utilizing the igraph package version 1.5.1 in R. The resulting network was then visualized using both the igraph package and Cytoscape version 3.10.1. The topological features of the networks were analyzed, including the count of both positive and negative correlations, as well as other network metrics such as betweenness centrality, closeness centrality, and eigenvector centrality.

### Microbial community-environment interaction analysis

The analysis involved exploring the connection between designated microbial communities in the intestine of *L. vannamei* and environmental samples, including sediment and water, along with water quality indicators. The vegan package version 2.6.4 in R was employed for this purpose. The primary focus was on the top 20 most abundant genera identified from both partial and full-length 16S rRNA gene data. To facilitate the analysis, the relative abundance of microbes at the genus level and water quality indicators underwent a log-transformation (lg(*x* + 1)), where *x* represents the relative abundance of each genus or water quality indicator. Canonical correspondence analysis (CCA) and redundancy analysis (RDA) were employed for ordination, guided by gradient lengths. Specifically, RDA was applied when gradient lengths were less than 3, either RDA or CCA when lengths fell between 3 and 4, and CCA when lengths exceeded 4. This approach aimed to uncover the relationship between microbial communities and water quality indicators.

## RESULTS

### Bacterial diversity in Ponds A (without) and B (with probiotic supplementation) across sample types

16S rRNA gene sequencing of shrimp intestine samples, respectively, yielded 1,677,453 long-reads and 2,652,155 short-reads across both platforms. Environmental samples included 642,998 long-reads and 1,492,455 short-reads from sediment, and 659,645 long-reads and 1,405,885 short-reads from water. Rarefaction curve analysis (Fig. S1) confirmed sufficient sequencing depth for both long- and short-reads, accurately capturing the bacterial community composition in all samples.

The Venn diagram revealed shared and unique microbial communities found in shrimp intestine, sediment, and water samples from Pond A (without probiotic supplementation) and B (with probiotics supplementation). A key group of taxa, in the central intersection, represents a core microbial community thriving in all three samples in Pond A or Pond B (Fig. S2). Interestingly, sediment samples, regardless of long-read or short-read sequencing, harbored the most diverse microbial inhabitants in both ponds. Moreover, there was a higher degree of overlap in bacterial communities between sediment and intestine samples (Fig. S2). This suggests a closer relationship and potential for frequent exchange of bacterial populations between the host shrimp and the surrounding sediments.

**Fig 1:**
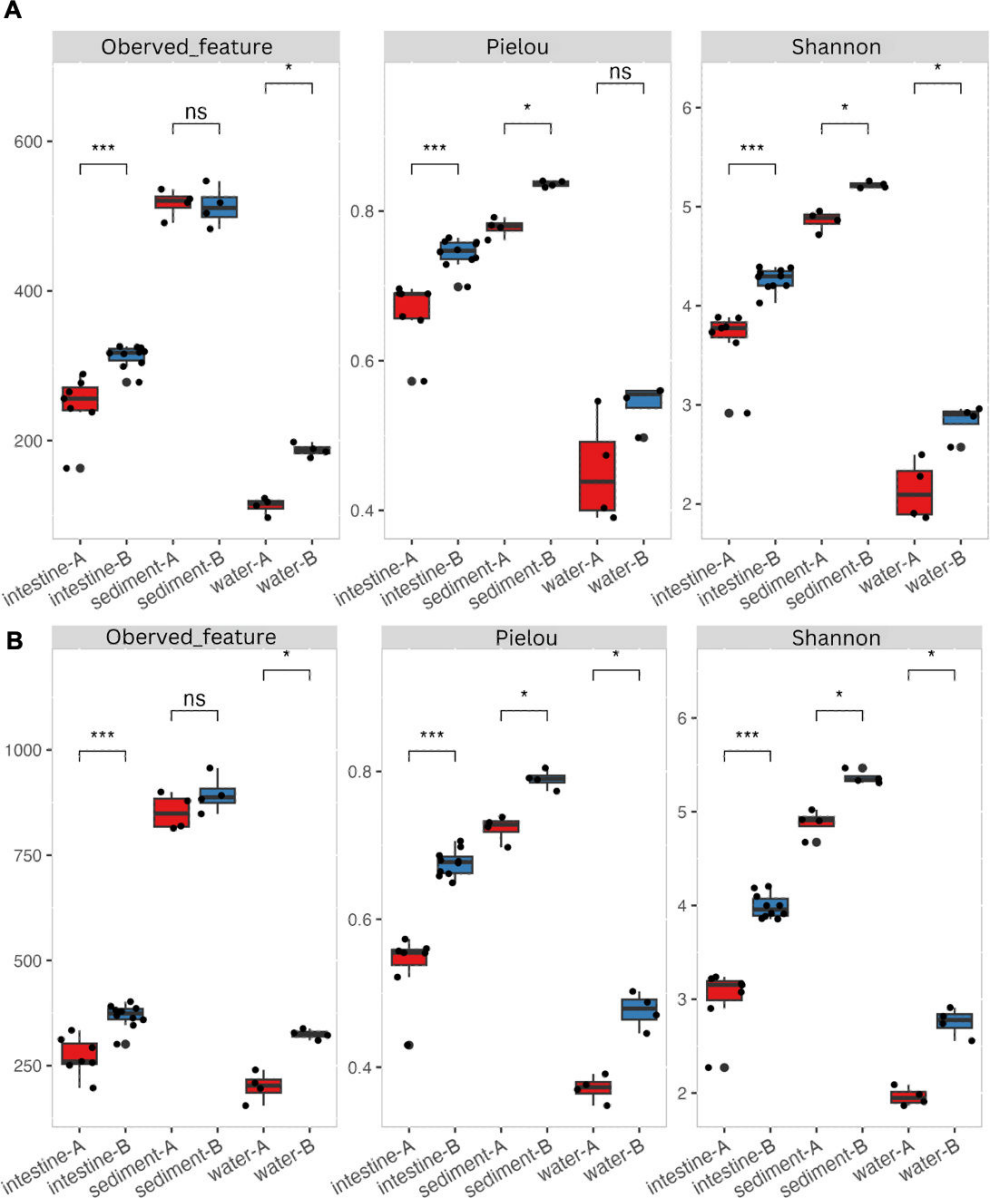
Comparative analysis of alpha diversity indices (observed species richness, Pielou, and Shannon diversity indices) in *L. vannamei* intestine and environmental samples (sediment and water) from Ponds A (without) and B (with probiotic supplementation) using long-reads (full-length 16S rRNA gene sequences) (**A**) and short-reads (partial 16S rRNA gene sequences) (**B**) 16S rRNA gene sequencing data. *, **, *** Significance at *P* < 0.01, 0.001, 0.0001. ns, non-significant.

Alpha diversity measures—observed species richness, Pielou, and Shannon diversity indices—were applied to evaluate bacterial community diversity in intestine, sediment, and water samples from Ponds A and B (Fig. 1). Both long- (Fig. 1A) or short-read sequencing (Fig. 1B) showed consistent patterns, in which Pond B showed significantly increased diversity (*P* < 0.05) in shrimp intestine, and water, except sediment sample when comparing to Pond A (Table 1). Pielou evenness index showed a similar pattern within water samples in the case of long-read sequencing (Table 1). Notably, both sequencing methods captured a consistent and statistically significant increase in the Shannon diversity index in Pond B compared to A for all sample types (Table 1). Furthermore, diversity indices, including Shannon and Pielou, exhibited significant variation among samples (*P* < 0.05). Sediment consistently contained the highest bacterial diversity, followed by the shrimp intestines. Water samples, on the other hand, hosted the least diverse communities of microbes (Fig. S3).

**TABLE 1:** Wilcoxon Rank-Sum test: alpha diversity variations in Ponds A (without) and B (with probiotic supplementation) across different sample categories (intestine, sediment, and water)^*a*^

16S rRNA gene data set	Observed species richness			Pielou index			Shannon index		
	Intestine	Sediment	Water	Intestine	Sediment	Water	Intestine	Sediment	Water
Long-read	0.000***	0.771 ns	0.029*	0.000***	0.029*	0.057 ns	0.000***	0.029*	0.029*
Short-read	0.000***	0.485 ns	0.029*	0.000***	0.029*	0.029*	0.000***	0.029*	0.029*

16S rRNA gene data	Pond A	Pond B	Pond A	Pond B	Pond A	Pond B
Long-read	0.002**	0.001**	0.002**	0.001**	0.002 **	0.001 **
Short-read	0.005**	0.002**	0.002**	0.001**	0.002 **	0.001 **

^*a*^ The asterisk (*) represents the *P*-value of the statistical test (*P < 0.01, **P < 0.001, and ***P < 0.0001). ns, non-significant.

**Fig 2:**
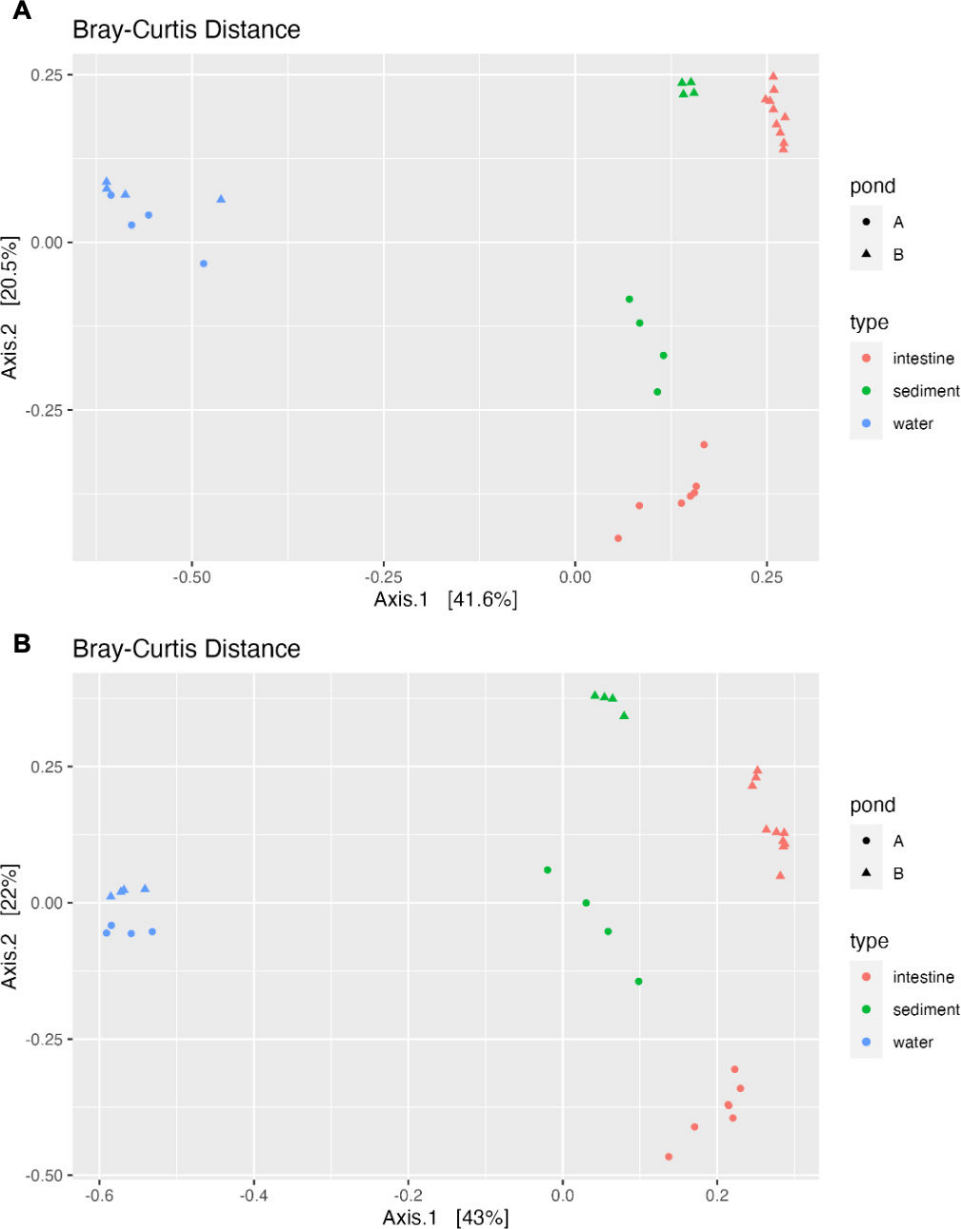
PCoA of bacterial community distribution and clustering analysis based on Bray-Curtis distance obtained from both long- (**A**) and short-read (**B**) sequencing results of *L. vannamei* intestine and environmental samples (sediment and water) from Ponds A (without) and B (with probiotic supplementation). Individual samples are marked as points, with differentiation by pond sources (A or B) indicated by shape, and sample categories (I = intestine, S = sediment, W = water) by color. The proximity of points reflects the degree of dissimilarity between samples, with closer clusters representing similar community structures.

To assess the similarity of bacterial communities in each sample, principal coordinate analysis (PCoA) was used to generate 2D visual maps. The PCoA plot, using Bray-Curtis’s distance, revealed distinct clusters for sediment, intestine, and water samples based on sample type (host shrimp, water, and sediment) (Fig. 2), indicating a stronger influence of habitat on bacterial composition. Water samples from Ponds A and B clustered closely, while sediment and intestine samples showed distinct clustering within their respective pond of origin, highlighting significant compositional differences associated with the pond sources.

The Adonis test was performed to further investigate species dissimilarities between samples from both ponds. Analyzing species dissimilarities revealed a substantial impact of both sample categories and pond sources on long-read sequencing data, explaining 82.5% of the variation (*R*^2^ = 82.5%) (Table 2). This suggests strong influence of both type of sample, 56% (e.g., water, sediment) and pond origin on microbial profiles in this case (Table 2). However, for short-read data, a significant effect was observed only for sample categories, explaining 38.9% of the variation (*R*^2^ = 38.9%). This implies that shorter reads may not capture the full extent of community differences between these samples. This highlights the potential influence of sequencing technology on deciphering microbial diversity and its underlying factors.

**TABLE 2:** Adonis test: bacterial community dissimilarities between pond sources and sample categories^*a*^

16S rRNA gene data set	Metric	Parameter	*R* ^2^	Pr (>*F*)	Significance
Long-read	Bray-Curtis distance	Pond sources	0.174	1e−04	***
		Sample categories	0.559 (55.9%)	1e−04	***
		Pond : samples	0.092	1e−04	***
		Total	0.825 (82.5%)		
Short-read	Bray-Curtis distance	Pond sources	0.023	0.377	ns
		Sample categories	0.279	0.000	***
		Pond : samples	0.087	0.064	ns
		Total	0.389 (38.9%)		

^*a*^ The asterisk (*) represents the *P*-value of the statistical test (***P < 0.0001). ns, non-significant.

### Comparison of bacterial community profiles in Ponds A (without) and B (with probiotic supplementation) across sample types from different sequencing platforms

#### Phylum-level analysis of bacterial communities

**Fig 3:**
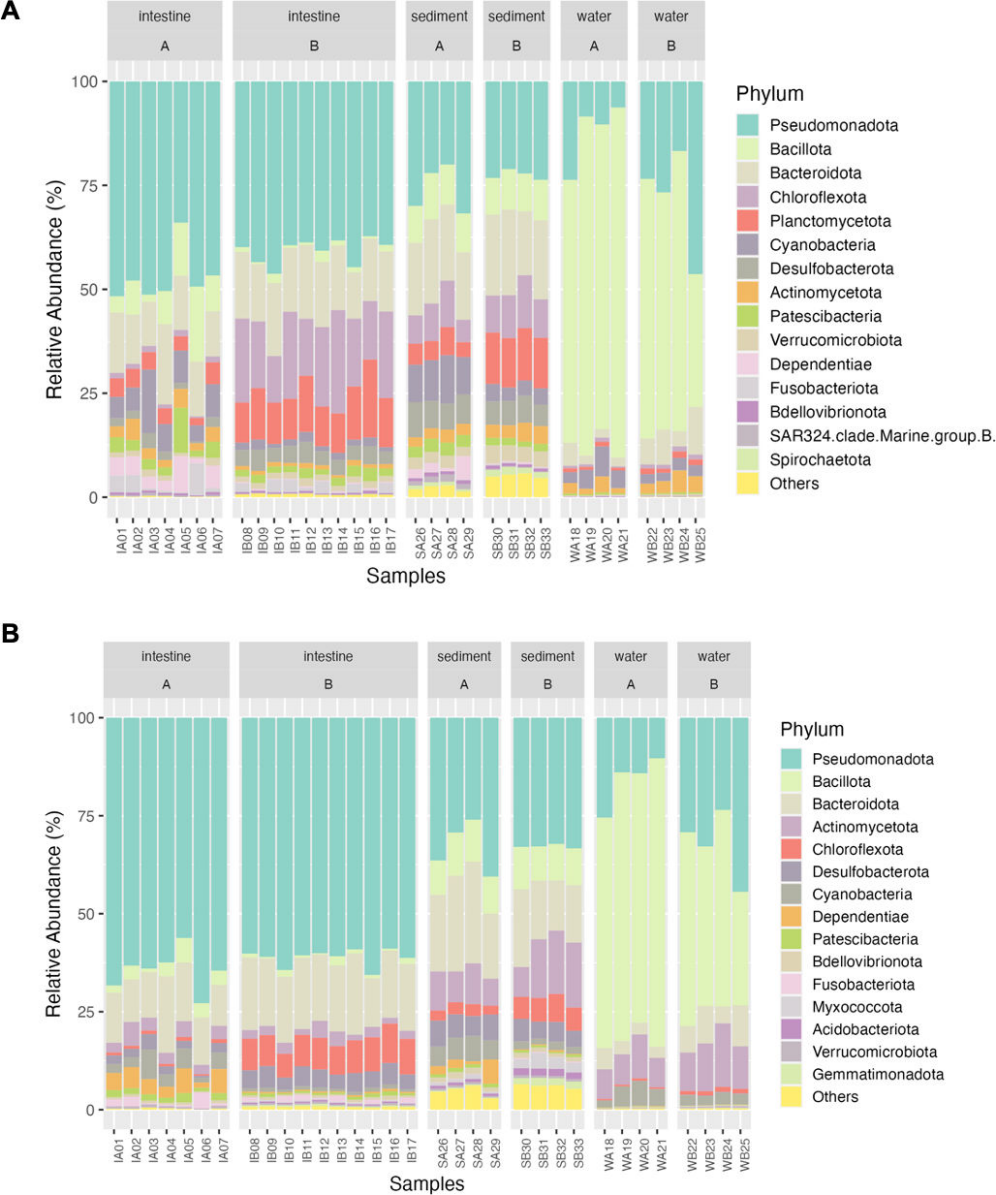
Comparative analysis of bacterial community structure in *L. vannamei* intestine and environmental samples (sediment and water) from Ponds A (without) and B (with probiotic supplementation): long-read (**A**) and short-read (**B**) 16S rRNA gene sequencing analysis at a phylum level.

Although their relative abundance varied between the two sequencing platforms (Table S2), the bacterial community profiles of *L. vannamei*, as revealed by both long-read (Fig. 3A) and short-read (Fig. 3B) sequencing, exhibited notable similarities. In both approaches, Pseudomonadota, Cyanobacteria, Bacteroidota, and Bacillota consistently remained the most abundant phyla in all sample types and among the top 20 most abundant phyla (Fig. 3).

Pseudomonadota was the most abundant phylum in both intestine (Pond A = 47%, Pond B = 40%, *P* < 0.05) and sediment samples (Pond A = 26%, Pond B = 22%, *P* > 0.05) (Table S2.1). Conversely, in water samples, Bacillota emerged as the top dominant phylum (Pond A = 75%, Pond B = 54%, *P* > 0.05) (Table S2.1). For intestine samples, Pond B differed from Pond A with a significant increase in abundance of Desulfobacterota, Chloroflexota, and Planctomycetota (*P* < 0.05), and a significant decrease in abundance of Pseudomonadota, Bacillota, Cyanobacteria, Actinobacteriota, and Dependentiae (*P* < 0.05) (Table S2.1). In sediment samples, a similar pattern emerged with Planctomycetota and Cyanobacteria, both showing significant increases and decreases in abundance (*P* < 0.05), respectively (Table S2.1). These findings emphasize the subtle distinctions in microbial composition among the samples and underscore the clear influence of environmental variations on the relative abundance of different phyla.

#### Genus-level analysis of bacterial communities

**Fig 4:**
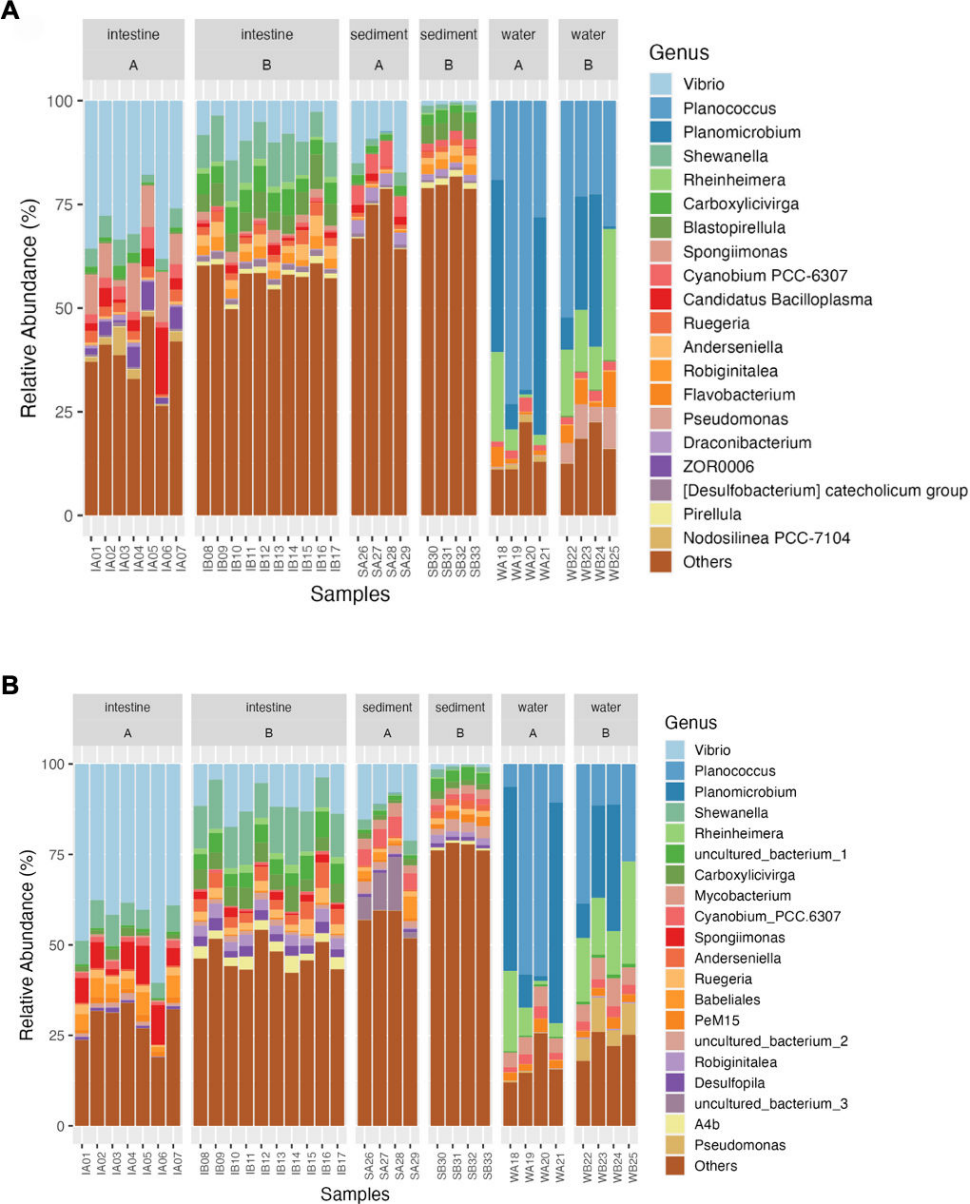
Comparative analysis of bacterial community structure in *L. vannamei* intestine and environmental samples (sediment and water) from Ponds A (without) and B (with probiotic supplementation): long-read (**A**) and short-read (**B**) 16S rRNA gene sequencing analysis at a genus level.

The results revealed significant differences between the top 20 genera identified through long-read and short-read sequencing methods (Fig. 4; Table S3). In long-read sequencing (Fig. 4A), eight genera were exclusively found among the top 20: *Blastopirellula, Candidatus* Bacilloplasma, *Flavobacterium*, *Draconibacterium,* ZOR0006, *Desulfobacterium catecholicum*, *Nodosilinea* PCC-7104, and *Pirellula*. In contrast, short-read sequencing (Fig. 4B) identified six different genera that were exclusive to its top 20: *Mycobacterium*, *Babeliales*, *PeM15*, *Desulfopila,* A4b, and uncultured_bacterium (Table S3.2). This shows that the choice of sequencing method impacts which genera are detected as the most abundant.

Intestine and sediment samples exhibited some key similarities. *Vibrio* was the dominant genus in the intestine samples, but its abundance significantly decreased in Pond B (Pond A = 30%, Pond B = 8%, *P* < 0.05) (Table S3.1). In addition, Pond B showed a difference in relative abundance for other genera. *Shewanella*, *Carboxylicivirga*, *Blastopirellula*, *Anderseniella*, and *Robiginitalea* were significantly increased (*P* < 0.05), while *Spongiimonas*, *Cyanobium* PCC-6307, *Ca*. Bacilloplasma, ZOR0006, and *Nodosilinea* PCC-7104 were significantly reduced. The sediment samples followed a similar pattern, with *Vibrio* as the primary genus but exhibiting a significant decline in Pond B (Pond A = 12%, Pond B = 0.85%, *P* < 0.05) (Table S3.1). Furthermore, there were substantial increases in relative abundance for *Blastopirellula* and *Robiginitalea* that together with *Blastopirellula*, they became the most abundant genera in Pond B. In contrast, *Cyanobium* PCC-6307 showed significantly lower abundance in Pond B (*P* < 0.05). Water samples displayed consistent top three genera (*Planococcus*, *Planomicrobium*, and *Rheinheimera*) with no significant differences between ponds (Table S3.1). *Pseudomonas*, another dominant genus, demonstrated a significant increase in abundance in Pond B in water samples (*P* < 0.05).

#### Species-level analysis of bacterial communities

**Fig 5:**
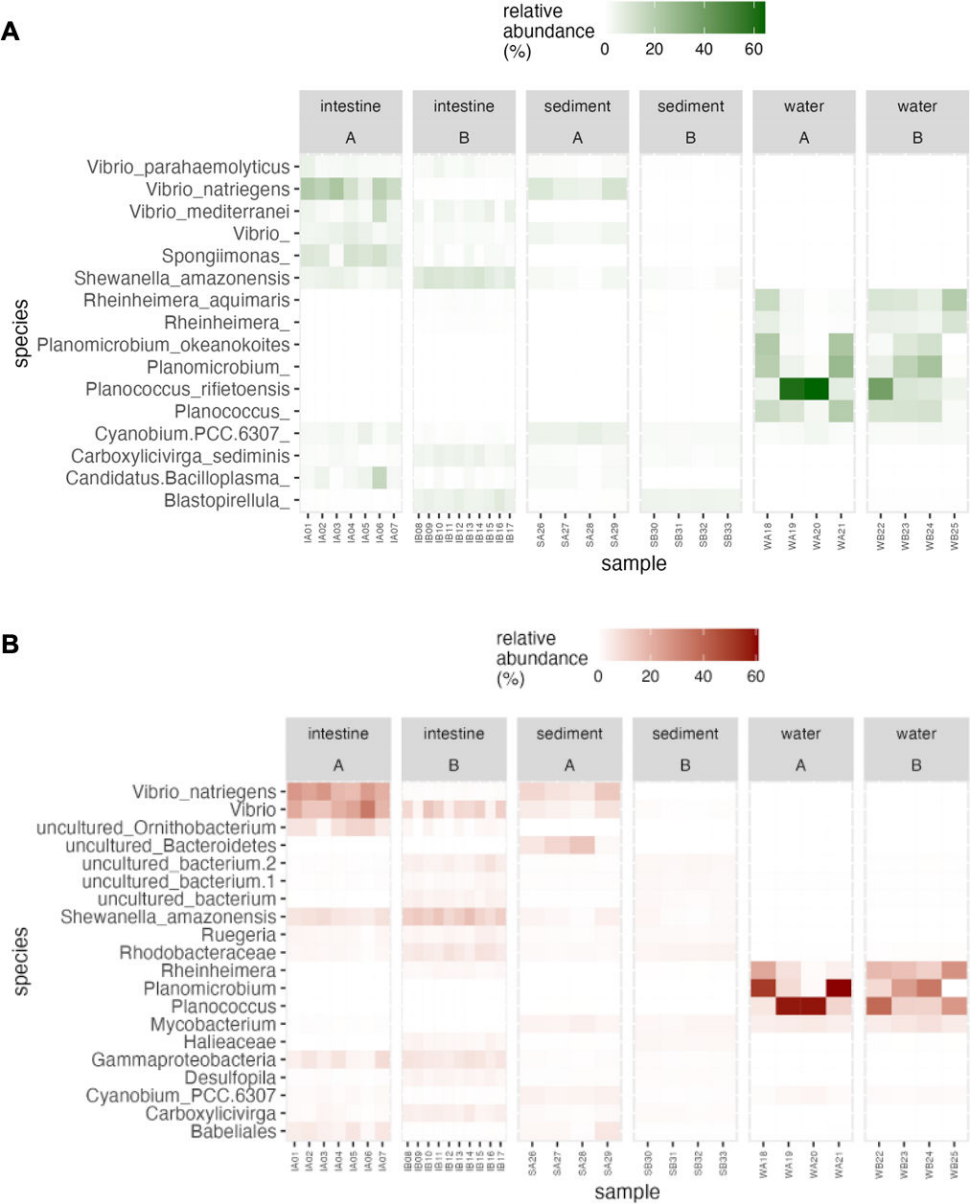
Comparative analysis of bacterial species abundance in *L. vannamei* intestine and environmental samples (sediment and water) from Ponds A (without) and B (with probiotic supplementation) utilizing long-read (**A**) and short-read (**B**) 16S rRNA gene sequencing: Heatmap visualization

Long-read sequencing (Fig. 5A) confirmed 10 fully identified species among the top 20 abundant species, while short-read sequencing (Fig. 5B) detected only three species (*Vibrio natriegens*, *Shewanella amazonensis*, *Cyanobium*_PCC-6307) (Table S4).

Three *Vibrio* species were identified: *V. natriegens*, *V. mediterranei,* and *V. parahaemolyticus*. In Pond A, *V. natriegens* was the most abundant species in intestine samples, followed by *Ca*. Bacilloplasma, *V. mediterranei*, *S. amazonensis*, *Cyanobium_*PCC-6307, *V. parahaemolyticus*, and *Carboxylicivirga sediminis* (Fig. 5A). In Pond B, there was a significant increase in the abundance of *S. amazonensis*, *C. sediminis,* and *Rheinheimera aquimaris*, while *V. natriegens*, *V. parahaemolyticus*, and *Cyanobium*_PCC-6307 significantly decreased (*P* < 0.05) (Table S4.1). In sediment samples, *V. natriegens* was the dominant species in Pond A, like the intestine samples, followed by *Cyanobium*_PCC-6307, *S. amazonensis*, *Ca*. Bacilloplasma, *C. sediminis*, and *V. parahaemolyticus* (Table S4.1). In Pond B, there was a significant decrease in the abundance of *V. natriegens*, *Cyanobium_*PCC-6307, and *Ca*. Bacilloplasma. *Cyanobium*_PCC-6307, although its abundance decreased in Pond B compared to Pond A, continued to be one of the most prevalent species alongside *C. sediminis* and *S. amazonensis* in Pond B. Water samples from both ponds exhibited the same species with no significant differences in their relative abundance. The most prevalent species, ranked from the highest abundance to the lowest, included *Planococcus rifietoensis*, *Planomicrobium okeanokoites*, *R. aquimaris*, and *Cyanobium_*PCC-6307 (Table S4.1).

Due to the better resolution for species identification, the full-length 16S rRNA gene sequences were chosen for the PICRUSt2 analysis at the species level and incorporating bacterial abundance values (Fig. S4). While no statistically significant differences in predicted metabolic pathways were observed between the intestinal microbiomes of shrimp from the two ponds (Fig. S4A), analysis of sediment and water samples revealed several metabolic pathways with statistically significant differences (Fig. S4B and C). The distinct metabolic pathways observed in sediment samples from Ponds A and B underscore the metabolic plasticity of bacterial communities in response to varying environmental conditions. These differences might reflect adaptations that enable bacteria to synthesize essential metabolites and efficiently utilize available nutrients. Water sample analysis demonstrated a statistically significant higher abundance of metabolic pathways in Pond B compared to Pond A (Fig. S4C). This suggests an enhanced metabolic potential in Pond B, which could potentially be driven by probiotic supplementation, with implications for increased substrate utilization and maintenance of essential cellular functions.

#### Differentially abundant species in Ponds A (without) and B (with probiotic supplementation) across sample types

**Fig 6:**
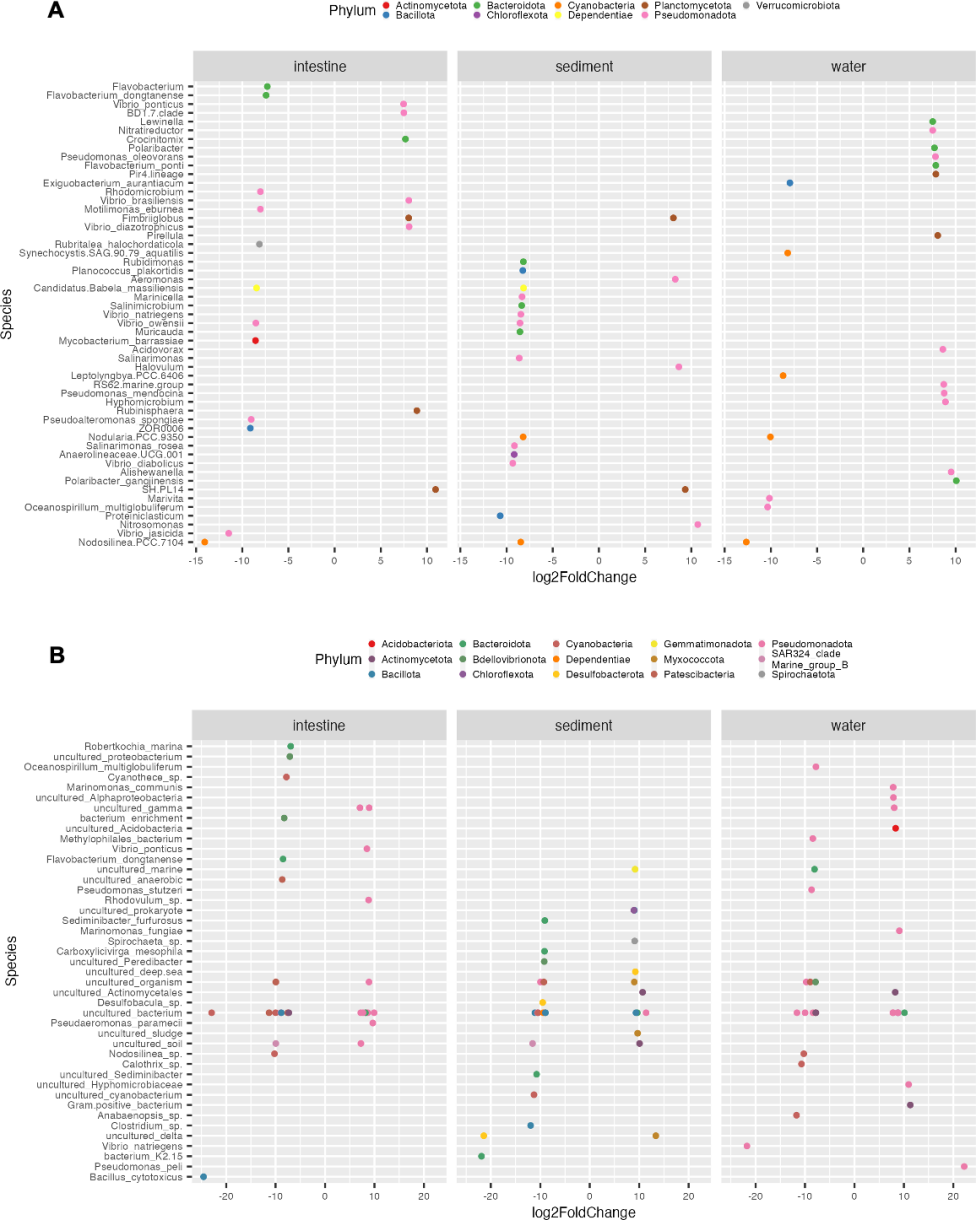
Comparative differential abundance analysis of bacterial species in *L. vannamei* intestine and environmental samples (sediment and water) from Ponds A (without) and B (with probiotic supplementation) using long-read (**A**) and short-read (**B**) 16S rRNA gene sequencing analysis of Log_2_ fold change visualization of differentially abundant species between ponds. The *y*-axis represents the log fold change values, while the *x*-axis represents the ponds. Positive values correspond to Pond A, whereas negative values indicate Pond B.

An extensive differential abundance analysis was performed to identify significant taxonomic disparities in the relative abundance of the top 20 species across sample categories within both ponds (Fig. 6). In long-read sequencing, in intestine samples, Pond A exhibited enrichments in *Vibrio brasilliensis* and *V. diazothophicus,* while Pond B displayed a broader range of enriched species, including *V. owensii*, *V. jasicida*, *Pseudoalteromonas spongiae*, *Mycobacterium barrassiae*, *Candidatus Babela* massilliensis, *Rubritalea halochordaticola*, and *Motillimonas ebumea* (Fig. 6A). For sediment samples, Pond A did not show any enriched species among the top 20 most abundant ones, while Pond B revealed enrichments in *V. diabolicus*, *Salinarimonas rosea*, *V. owensii*, and *V. natriegens* (Fig. 6A). In water samples, Pond A demonstrated enrichments in *Flavobacterium ponti*, *Pseudomonas mendocina*, and *Polaribacter gangjinensis*. On the other hand, Pond B displayed enrichments in *Oceanospirillum multiglobuliferum*, *Synechocystic*.SAG.90.79 *aquatilis*, and *Exiguobacterium aurantiacum* (Fig. 6A).

In short-read sequencing, several species were enriched in intestine samples from both ponds. Pond A exhibited enrichments in *Pseudoaeromonas paramecii* and *Vibrio ponticus*, while Pond B displayed enrichments in *Flavobacterium dongatanense*, *Robertkochia marina*, and *Bacillus cytotoxicus* (Fig. 6B). Remarkably, sediment samples obtained from Pond A exhibited no enriched species, in contrast to Pond B, where *Carboxylicivirga mesophila*, *Sediminibacter furfurosus*, and *Oceanispirochaeta litoralis* were notably enriched (Fig. 6B). For water samples, Pond A demonstrated enrichments in *Pseudomonas peli* and *Marinomonas fungiae*, while Pond B showed enrichments in *Vibrio natriegens*, *Pseudomonas stutzeri*, and *Oceanospirillum_multiglobuliferum* (Fig. 6B). These enriched species, except *Oceanispirochaeta litoralis*, belonged to Pseudomonadota, Bacillota, and Bacteroidota phyla, consistent with the prevailing phyla identified in the current microbial communities of *L. vannamei* culture ponds.

### Effect of environmental factors on bacterial community

Due to enhanced resolution for microbial profiles, the full-length 16S rRNA gene sequences were chosen for further investigation to elucidate the influence of environmental variables on bacterial communities associated within distinct habitats, specifically, the shrimp intestine, sediment, and water, from Ponds A (without) and B (with probiotic supplementation). Environmental parameters were routinely measured from the rearing water in both ponds (Table S5.1). The ordination models were chosen based on the outcomes of Detrended Correspondence Analysis (DCA) (Table S5.2). The genus-environment correlation in Redundancy Analysis (RDA) provided insights into the variance explained by each ordination axis in relation to the genus, with cumulative percentage variance detailing the explained variance of environmental variables concerning the genus (Table S5.3).

**Fig 7:**
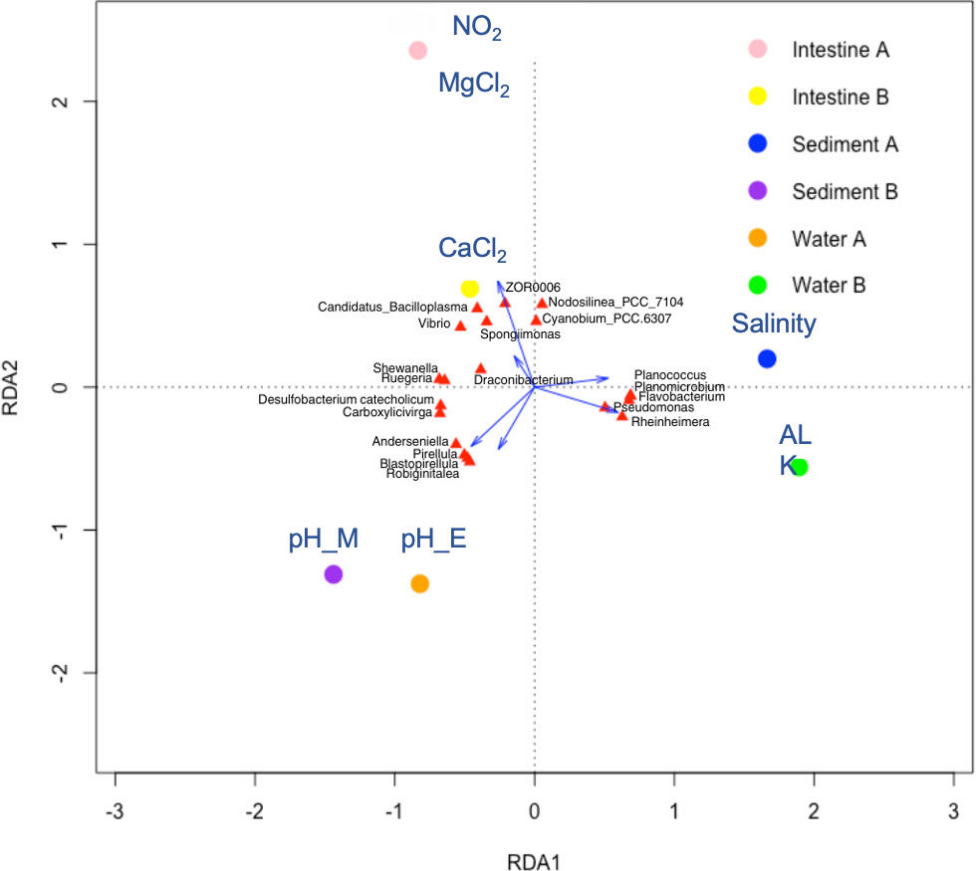
RDA of water quality parameters and the top 20 genera: exploring relationships and microbiome origins in Ponds A (without) and B (with probiotic supplementation) using long-read 16S rRNA gene sequencing data. Water quality parameters (CaCl_2_, calcium chloride; MgCl_2_, magnesium chloride; salinity; pH_M, morning pH; pH_E, evening pH; ALK, alkalinity; and NO_2_, nitrogen dioxide) are represented by arrows. The top 20 most-abundant genera are organized by sample categories and pond sources (A or B). Filled circle symbols denote the origin of the microbiome.

The first ordination axis exhibited a positive correlation with both ALK and salinity, while morning pH showed the most negative correlation. The genus–environment correlation coefficient for the first ordination axis was 11.22 with cumulative percentage variance of the genus-environment relation at 56% (Table S5.3). Therefore, an increase in ALK level and a decrease in pH were associated with enhanced diversity and abundance of bacterial species in water and sediment samples. The second ordination axis demonstrated positive correlations with both MgCl_2_ and NO_2_ content and negative correlations with morning pH. Sediment sample from Ponds A and water samples from Pond B exhibited a distinct correlation with the rearing environment, notably in relation to ALK and salinity, as illustrated in the ordination diagram (Fig. 7). This indicates a correlation between bacterial communities and these specific environmental factors. A similar pattern was observed for sediment sample from Pond B and water samples from Pond A, where their positions in the ordination diagram were associated with pH. Intestine samples from both ponds positively correlated with CaCl_2_, MgCl_2_, and NO_2_.

Correlation analysis focusing on the top 20 bacterial genera and environmental factors (Fig. 7) revealed varying adaptabilities to the rearing environment of varying qualities. Some bacterial genera displayed positive correlations with both salinity and ALK, including *Planoccocus*, *Planomicrobium*, *Flavobacterium*, *Pseudomonas*, and *Rheinheimera*. Conversely, others displayed negative correlations with pH, such as *Anderseniella*, *Pirellula*, *Blastopirellula*, *Robiginitalea*, *Carboxylicivirga*, and *Desulfobacterium catecholicum. Vibrio*’s abundance showed a strong positive correlation with MgCl_2_, and NO_2_, and a negative correlation with pH. Similar correlation patterns with the environmental factors were observed for *Candidatus* Bacilloplasma, *Spongimona*, ZOR0006, and *Draconibacterium*, echoing the observation for *Vibrio*.

### Network analysis of bacterial communities in Ponds A (without) and B (with probiotic supplementation) across sample types

**Fig 8:**
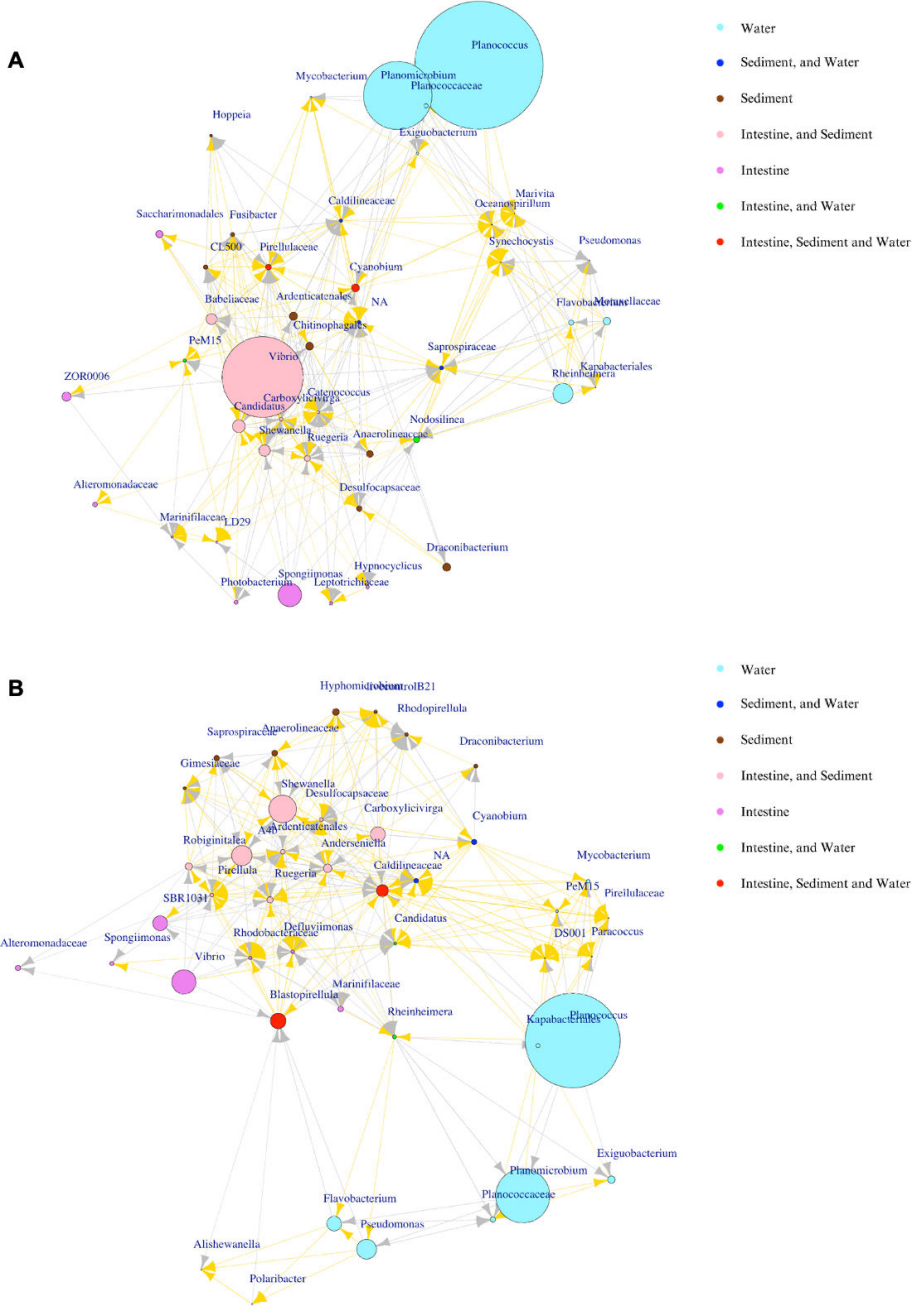
Correlation-based network analysis of the top 20 genera in Pond A (without) (**A**) and Pond B (with probiotic supplementation) (**B**) across shrimp intestine and environmental samples using long-read 16S rRNA gene sequencing data. Node color indicates sample source: violet (intestine), cadet blue (water), brown (sediment), blue (genera identified in both environmental samples), pink (genera identified in both intestine and sediment samples), red (genera identified in all sample categories), and green (genera identified in intestine and sediment samples). Node size reflects genus relative abundance. Correlation lines colored in gold for positive correlation and in gray for negative correlation.

A co-occurrence network, established using Spearman’s correlation, was employed to examine the relationships between intestine and environmental samples from both Ponds A and B. The networks unveiled a highly interconnected bacterial community, characterized by a prevalence of positive correlations over negative ones, linking intestine and environmental samples from either Pond A or Pond B (Fig. 8; Table S6.1). No discernible disparity in the genus interaction network was observed between Ponds A and B. All networks exhibited a clustering of bacterial communities from intestine and sediment samples, while those from the water were distinctly separated from other bacterial communities.

*Carboxylicivirga* exhibited the highest betweenness centrality values of 109.18 nodes in Pond A and *Reinheimera* with 337.41 nodes in Pond B (Table S6.2). Betweenness centrality quantifies the extent to which a node lies on the shortest paths between other nodes in the network. Regarding closeness centrality, which measures the average distance from a given genus to all other genera in the network, *Pseudomonas* ranked highest with a value of 1 in Pond A (Table S6.2). In Pond B, *Caldilineaceae* and *Carboxylicivirga* shared the highest closeness centrality values of 0.014 and 0.013, respectively. Within Pond A, *Planococcus*, *Cyanobium*, and *Draconibacterium* demonstrated the lowest closeness centrality values, ranging from 0.012 to 0.015. Meanwhile, in Pond B, *Spongiimonas*, *Draconibacterium*, LivecontrolIB21, *Desulfocapsaceae*, and *Rhodopirellula* exhibited the lowest closeness centrality values, ranging from 0.004 to 0.005. Eigenvector centrality, measuring the relative importance of each node based on its connections to highly connected nodes, identified *Carboxylicivirga* as pivotal with values of 1 in Pond A and *Caldilineaceae* with the highest values of 1 in Pond B (Table S6.2).

## DISCUSSION

Growing evidence emphasizes a strong connection between shrimp rearing environments and microbial diversity (4, 11, 12, 20, 35, 36). It is well-established that technical factors can notably influence the outcomes of bacterial community profiling. To examine the structure, diversity, and dynamics of microbial populations in both *L. vannamei* culture ponds and the shrimp intestine, we used both full-length and partial 16S rRNA gene sequences. Our results revealed a core bacterial community shared across shrimp intestine, sediment, and water samples from both Ponds A (without) and B (with probiotic supplementation), suggesting key roles in maintaining ecosystem stability. The dynamics of this core community appear to be fine-tuned by an interplay of farming practices, environmental factors, and specific ecological niches.

### Exploring bacterial community sequencing approaches

Short-read sequencing, a recent advancement in high-throughput sequencing, has emerged as a widely used method for microbial taxonomy profiling, alongside traditional methods such as Denaturing Gradient Gel Electrophoresis (DGGE) (13–15, 22). Technical disparities in short-read sequencing involve the selection of the amplified 16S rRNA gene hypervariable region (V1-V9) (37), a consideration not applicable to long-read sequencing. Alpha diversity, however, remained unaffected by sequence length. The observed species richness and Shannon index consistently show similar results for both short- and long-read sequencing methods, highlighting their robustness in alpha diversity analysis irrespective of the sequencing approach. Additionally, the Shannon index was previously found to yield consistent species diversity estimates despite differences in the hypervariable regions of the 16S rRNA gene used (38). Beta diversity exhibited some sensitivity to sequencing length, as evidenced by the adonis test (Table 2). Species dissimilarities were influenced by both pond sources and sample categories in long-read sequencing but only by sample categories in short-read sequencing. Previous studies have also indicated the potential impact of sequencing length on beta diversity (39). Nevertheless, the clustering pattern, as revealed by PCoA plots, remained similar between the sequencing methods.

While short- and long-read sequencing exhibited high similarity in phyla identification, notable differences emerged in bacterial relative abundances. A study on mouse intestinal microbiota similarly observed such differences between short- and long-reads (39), mirroring our findings. In our study, Pseudomonadota dominated intestine samples, constituting 47% and 64% of the community for short- and long-reads, respectively, in Pond A, and 40% and 61% in Pond B. Beyond relative abundance, discrepancies extended to lower-rank taxa (genera and species), with long-reads consistently identifying more genera and species in both ponds. Distinct taxa were also found exclusively in either short- or long-reads.

Our study revealed significant differences in microbial identification between long- and short-read sequencing, particularly at the species level. Notably, long-reads identified a higher number of *Vibrio* species among the top 20 most abundant, including disease indicators in shrimp farming, such as *V. parahaemolyticus* and other group, *Candidatus* Bacilloplasma (40–43). Conversely, short-reads uniquely identified genera like PeM15, uncultured-bacterium, and *Mycobacterium* (42, 44, 45), previously found in shrimp farming but not as abundant with long-reads.

These contrasting results emphasize the impact of sequencing methodology on microbial identification. Consistent with our findings, previous research has shown variations within short-read data due to targeted 16S rRNA regions and primer selection (38), impacting shrimp microbiome profiling. Similar discrepancies have been observed in human gut microbiota studies using 16S full‐length‐based synthetic long‐read (sFL16S) and V3–V4 short‐read methods (26). The complementary data of long- and short-read sequencing suggest a tailored approach for microbial profiling in shrimp farming. Short-read sequencing would be sufficient for routine profiling, while long-read sequencing will be more suitable for precise strain definition or differentiation between closely related strains in specific niches.

### Bacterial community dynamics in Ponds A and B and across samples

Microbial communities in Ponds A and B exhibited distinct dynamics across water, sediment, and intestine samples, influenced by environmental factors. Salinity and alkalinity (ALK) were key drivers in water and sediment, while calcium chloride (CaCl_2_), magnesium chloride (MgCl_2_), and nitrite (NO_2_) impacted intestinal bacteria in shrimp. Notably, salinity predominantly affected the water microbiome, aligning with previous research (7, 20). The pH and ALK values were negatively correlated in sediment and water, highlighting their role in maintaining aquatic ecosystem stability (46). Hence, managing these factors could potentially control the abundance of certain bacterial genera, such as *Planococcus*, *Planomicrobium*, *Flavobacterium*, *Pseudomonas*, and *Rheineimera* aligning with strategies for maintaining microbial balance management in shrimp (7, 47).

Our findings also showed the dominance of Pseudomonadota in *L. vannamei* intestines, consistent with previous studies, and the abundance of *Rheinheimera aquimaris* in water samples (13–15, 22–25). Additionally, while Bacillota are commonly found in intestine of *L. vannamei* and other penaeid shrimp, they were primarily observed in water samples in this study, with *Planococcus* and *Planomicrobium* being the dominant genera. Given the dynamic nature of pond environments, an understanding of the interplay between environmental factors and microbial communities in shrimp ponds, particularly through long-read sequencing, is crucial for optimizing shrimp health and productivity in aquaculture.

### Bacterial predicted functional potentials and community networks and in Ponds A and B across sample types

There were no significant differences in predicted metabolic pathways within shrimp gut microbiomes suggesting that probiotic supplementation or environmental factors might not be the keys to alter the core metabolic functions of these communities. This could be due to the stability of the gut microbiome due to the host selective pressures (48). However, the significant differences observed in sediment and water microbiomes indicated that environmental bacteria were more susceptible to modulation by external factors, such as probiotic introduction. The distinct metabolic pathways observed in Pond B’s sediment, including those related to nutrient utilization, suggesting that the probiotic supplementation might provide positive impact on adaptable microbial community. This could have implications for nutrient cycling, disease resistance, and overall pond health. While the findings suggested that probiotic supplementation might influence functional differences between the ponds, further experimental validation will be required to confirm these predictions.

Network analysis revealed a complex ecological network with bacterial communities exhibiting predominantly positive correlations, suggesting cooperative relationships between intestine, water, and sediment samples. The heightened clustering in the network of the bacterial communities from the intestine and sediment samples indicated greater similarities in their bacterial compositions compared to those found in water samples, as also evidenced by their beta diversity. This observation suggests a robust connection and substantial mutual influence between the bacterial communities in the intestine and sediment.

Network analysis revealed key genera influencing bacterial community dynamics in shrimp ponds. In Pond A, *Carboxylicivirga* was central to network connectivity, while in Pond B, *Caldilineaceae* and *Rheinheimera* played similar roles. *Carboxylicivirga*, sensitive to environmental factors like pH and salinity (49, 50), was enriched in probiotic-treated sediment, potentially contributing to organic matter decomposition. *Rheinheimera*, positively correlated with alkalinity and salinity, is known to be highly responsive to environmental changes (51).

### Conclusion

Short- and long-read sequencing methods were compared for bacterial community profiling. While both methods showed similar alpha diversity, beta diversity exhibited some sensitivity to sequencing length, with long-reads capturing more differences. Short-read sequencing is cost-effective but limited to genus-level resolution, while long-reads offer species-level accuracy at the expense of higher error rates. Long-reads identified more taxa and disease indicators, while short-reads detected exclusive genera. Both methods complement each other, with short-reads suitable for routine profiling and long-reads for precise strain definition.

### Highlights

- This work represents a field study on the microbiome of the Pacific white shrimp intestine and their rearing environment
- Sequence length impact on accurate microbial profiling in shrimp ponds was assessed.
- Ecological niches explain 56% of bacterial variations in culture ponds.
- Consistent taxa in full-length 16S rRNA gene and V3–V4 support short-reads in shrimp microbiome study.

## Data Availability

The sequencing data generated under this study have been deposited at the National Center for Biotechnology Information (NCBI), BioProject accession number PRJNA1087723.

## SUPPLEMENTAL MATERIAL

Fig. S1 spectrum.00965-24-s0001.docx Alpha diversity rarefaction curve analysis with long-read and short-read 16S rRNA sequencing data.

Fig. S2 spectrum.00965-24-s0002.docx Venn diagram analysis of unique and shared species among samples from ponds A and B.

Fig. S3 spectrum.00965-24-s0003.docx Comparative analysis of alpha diversity indices.

Fig. S4 spectrum.00965-24-s0004.docx Clustered bar chart showing the differentiated putative metabolic pathways of the bacterial communities between pond A and pond B.

Supplemental tables spectrum.00965-24-s0005.xlsx Tables S1 to S6.

ASM does not own the copyrights to Supplemental Material that may be linked to, or accessed through, an article. The authors have granted ASM a non-exclusive, world-wide license to publish the Supplemental Material files. Please contact the corresponding author directly for reuse.
