# Supplementary material for: Integrating short- and full-length 16S rRNA gene sequencing to elucidate microbiome profiles in Pacific white shrimp (Litopenaeus vannamei) ponds
Source: Microbiol Spectr. 2024 Sep 27;12(11):e00965-24. doi: 10.1128/spectrum.00965-24 (PMC11537064; doi:10.1128/spectrum.00965-24)
Supplement: Fig. S1 — Alpha diversity rarefaction curve analysis with long-read and short-read 16S rRNA sequencing data. [file spectrum.00965-24-s0001.docx]

A


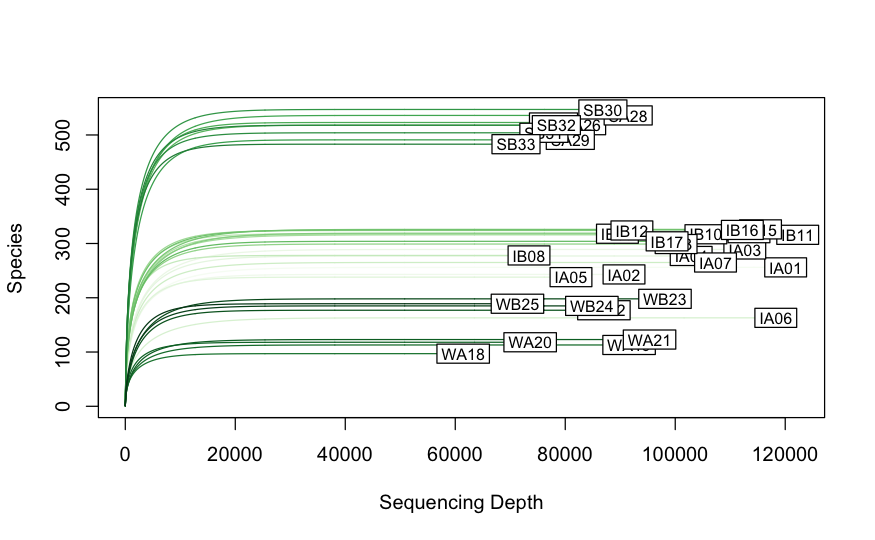


B


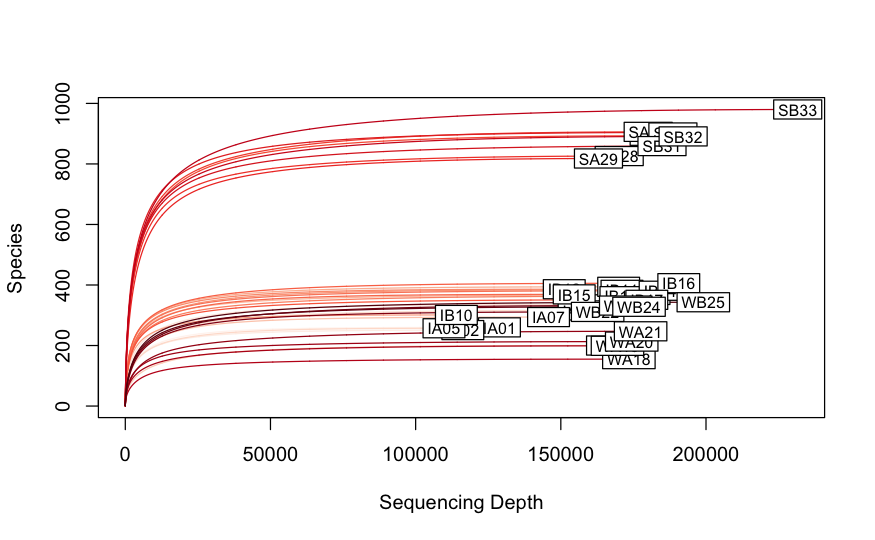


**Figure S1.** (A) Alpha Diversity Rarefaction Curve Analysis with Long-Read **(A)** and Short-Read **(B)** 16S rRNA Sequencing Data. The X-axis represents sequencing sampling depth, and the Y-axis represents species count. Samples are coded to distinguish between categories (I=intestine, S=sediment, W=water) and pond sources (A=pond A, B=pond B) with unique identifier number.
