# Supplementary material for: Integrating short- and full-length 16S rRNA gene sequencing to elucidate microbiome profiles in Pacific white shrimp (Litopenaeus vannamei) ponds
Source: Microbiol Spectr. 2024 Sep 27;12(11):e00965-24. doi: 10.1128/spectrum.00965-24 (PMC11537064; doi:10.1128/spectrum.00965-24)
Supplement: Fig. S2 — Venn diagram analysis of unique and shared species among samples from ponds A and B. [file spectrum.00965-24-s0002.docx]

A


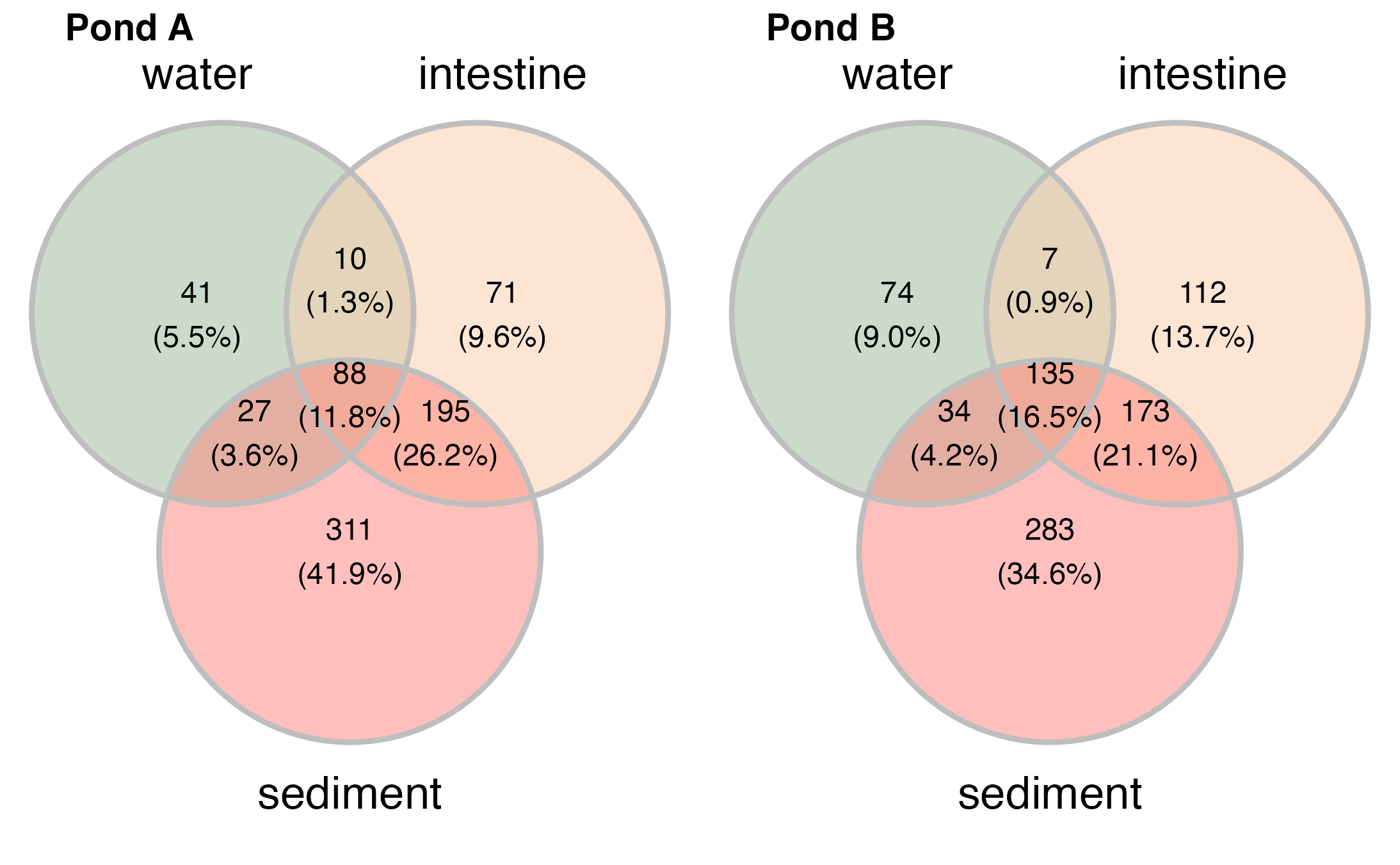


B


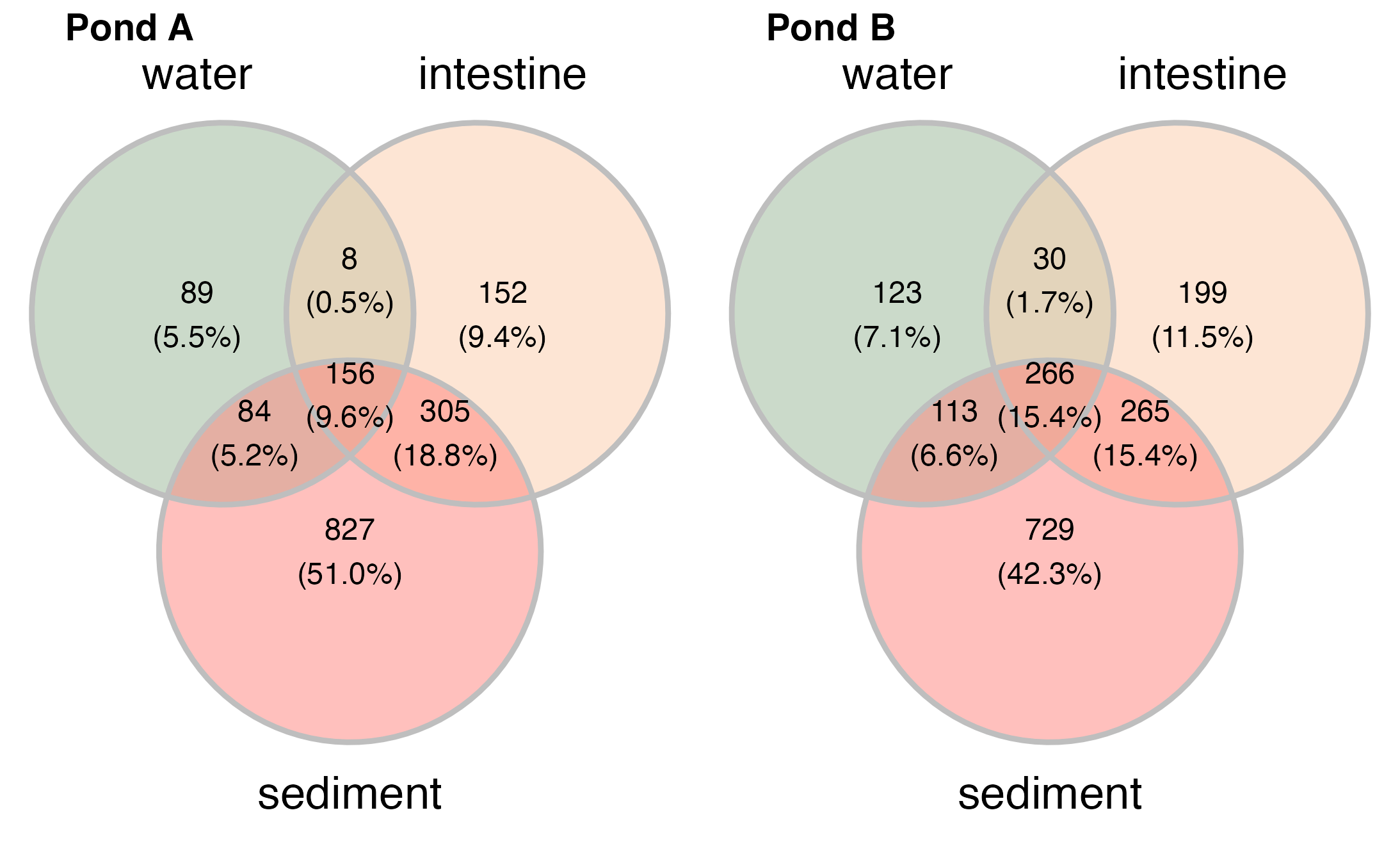


**Figure S2.** Venn Diagram Analysis of Unique and Shared Species among Samples from Ponds A and B Using Long-Read **(A)** and Short-Read **(B)** 16S rRNA Sequencing Data.
