# Supplementary material for: Integrating short- and full-length 16S rRNA gene sequencing to elucidate microbiome profiles in Pacific white shrimp (Litopenaeus vannamei) ponds
Source: Microbiol Spectr. 2024 Sep 27;12(11):e00965-24. doi: 10.1128/spectrum.00965-24 (PMC11537064; doi:10.1128/spectrum.00965-24)
Supplement: Fig. S3 — Comparative analysis of alpha diversity indices. [file spectrum.00965-24-s0003.docx]

A


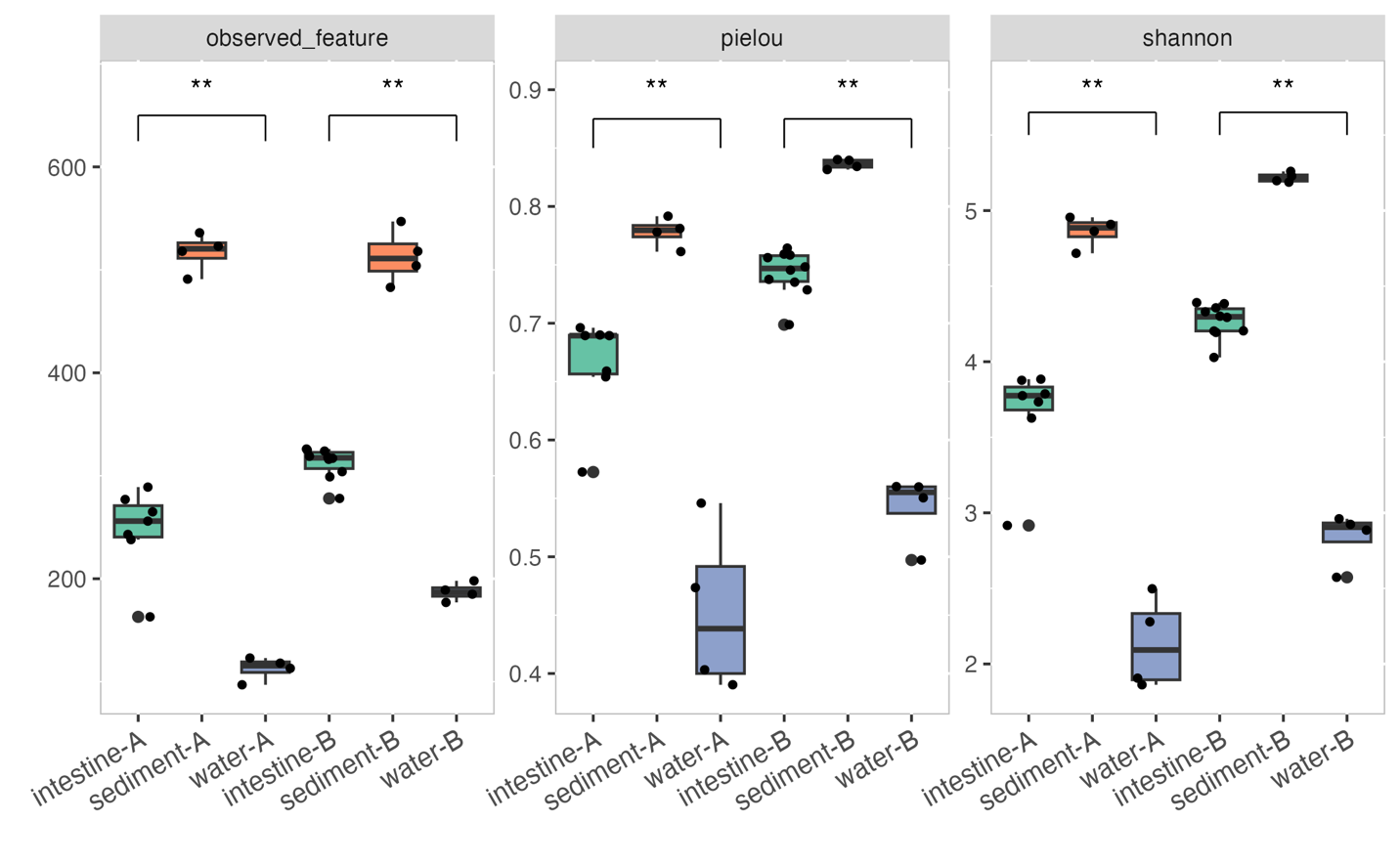


B


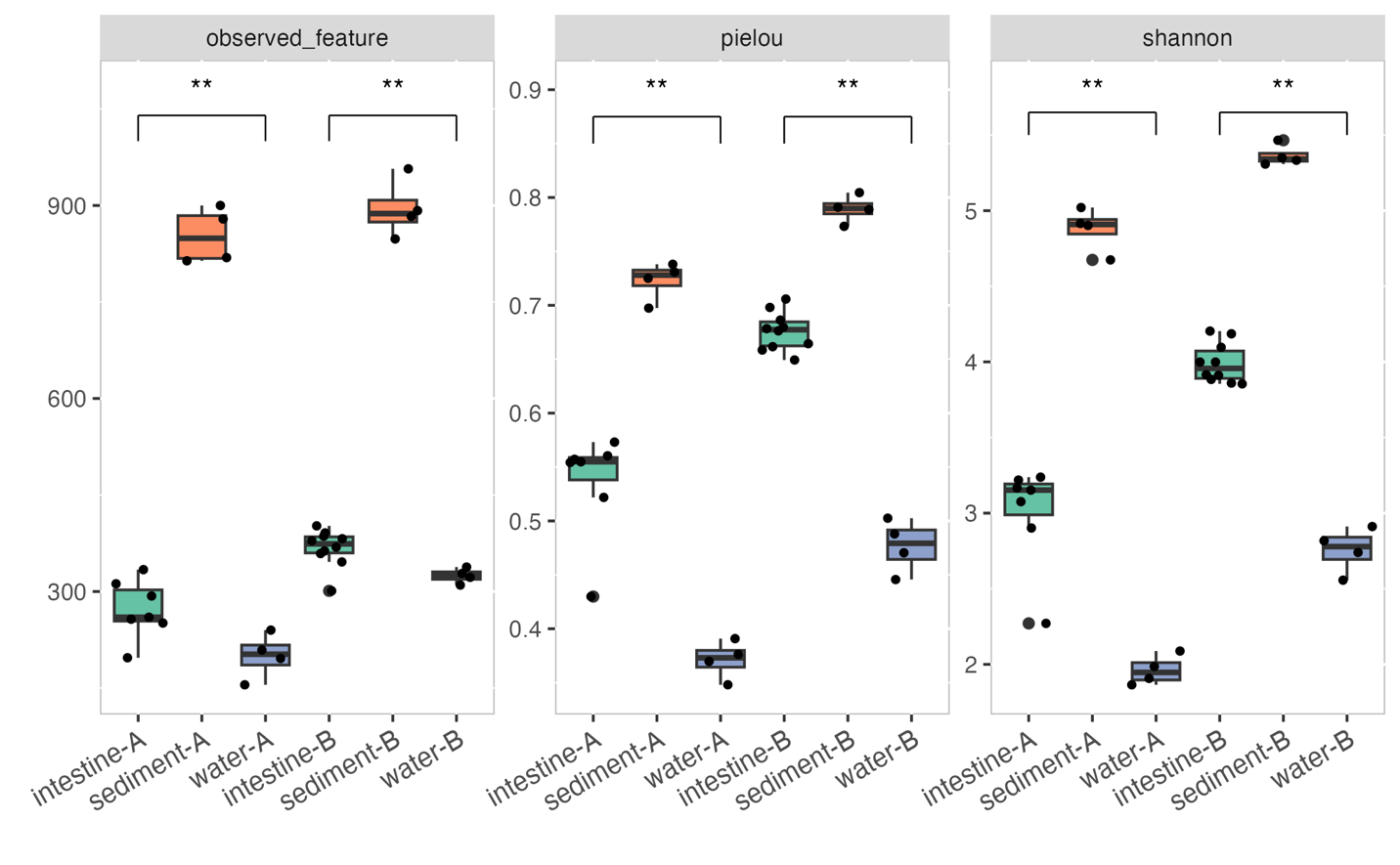


**Figure S3.** Comparative Analysis of Alpha Diversity Indices (Observed Species Richness, Pielou and Shannon Diversity indices) between Sample Categories (Intestine and Environmental Samples) from Pond A (without) and B (with probiotic supplementation) Using Long-Read **(A)** and Short-Read **(B)** 16S rRNA Sequencing Data. *, ** Significance at p< 0.01, 0.001. ns: Non-significant.
