# Supplementary material for: Integrating short- and full-length 16S rRNA gene sequencing to elucidate microbiome profiles in Pacific white shrimp (Litopenaeus vannamei) ponds
Source: Microbiol Spectr. 2024 Sep 27;12(11):e00965-24. doi: 10.1128/spectrum.00965-24 (PMC11537064; doi:10.1128/spectrum.00965-24)
Supplement: Fig. S4 — Clustered bar chart showing the differentiated putative metabolic pathways of the bacterial communities between pond A and pond B. [file spectrum.00965-24-s0004.docx]

A

B

C

**Figure S4.** Clustered bar chart showing the differentiated putative metabolic pathways of the bacterial communities between Pond A (without probiotic supplementation) and Pond B (with probiotic supplementation) within the sample categories (**A:** Intestine, **B:** Sediment, and **C:** Water) as predicted by the Phylogenetic Investigation of Communities by Reconstruction of Unobserved States (PICRUSt2) analysis. Rows represent the differentiated pathways from MetaCyc database (adjusted p < 0.05), and the bars in the graph represent the relative abundance of the functional categories. The log2 fold change reflects the difference in relative abundance, and adjusted p-values are displayed on the right of the figure.
